# Barriers and facilitators to using a clinical decision support tool for the management of osteoarthritis pain in patients undergoing hemodialysis: a qualitative study

**DOI:** 10.1186/s12875-024-02564-5

**Published:** 2024-08-19

**Authors:** Mai Mohsen, Angelina Abbaticchio, Tracy Zhang, S Vanita Jassal, Marisa Battistella

**Affiliations:** 1 Leslie Dan Faculty of Pharmacy, University of Toronto, Toronto, ON Canada; 2 Department of Nephrology, Toronto General Hospital – University Health Network, Toronto, ON Canada; 3 Medicine, University of Toronto, Toronto, ON Canada; 4 Department of Pharmacy, University Health Network, 200 Elizabeth St. EB 214, Toronto, ON M5G 2C4 Canada

**Keywords:** Hemodialysis, Osteoarthritis, Pain management, Barriers, Facilitators, Theoretical Domains Framework

## Abstract

**Background:**

While osteoarthritis is a significant issue within the hemodialysis population and contributes to reduced quality of life, pain related to osteoarthritis is poorly managed by healthcare professionals (HCPs) in hemodialysis settings due to the absence of clinical guidance applicable to this population. The purpose of this study was to explore the perceptions of HCPs on the barriers and facilitators to using a clinical decision support tool for osteoarthritis pain management in the hemodialysis setting.

**Methods:**

A qualitative descriptive study was conducted. Purposeful and snowball sampling techniques were used to recruit hemodialysis clinicians from academic and community settings across multiple Canadian provinces. One-to-one interviews were conducted with clinicians using a semi-structured, open ended interview guide informed by the Theoretical Domains Framework, a behavior change framework. A general inductive approach was applied to identify the main themes of barriers and facilitators.

**Results:**

A total of 11 interviews were completed with 3 nephrologists, 2 nurse practitioners and 6 pharmacists. Findings revealed 6 main barriers and facilitators related to the use of the clinical decision support tool. Alignment of the tool with practice roles emerged as a key barrier and facilitator. Other barriers included challenges related to the dialysis environment, varying levels of clinician comfort with pain medications, and limited applicability of the tool due to patient factors. An important facilitator was the intrinsic motivation among clinicians to use the tool.

**Conclusions:**

Most participants across the included hemodialysis settings expressed satisfaction with the clinical decision support tool and acknowledged its overall potential for improving osteoarthritis pain management among patients on hemodialysis. Future implementation of the tool may be limited by existing roles and practices at different institutions. Increased collaboration among hemodialysis and primary care teams may promote uptake of the tool.

**Supplementary Information:**

The online version contains supplementary material available at 10.1186/s12875-024-02564-5.

## Introduction

Osteoarthritis is a significant cause of suffering for patients with end stage kidney disease undergoing hemodialysis [1–5]. Chronic pain related to osteoarthritis leads to functional limitations, disability, social isolation, sleep disorders and depression which is an independent risk factor of mortality in the hemodialysis population [6–8]. Despite these negative health effects, pain related to osteoarthritis is poorly managed by healthcare professionals (HCPs) in hemodialysis settings due to the absence of clinical guidance applicable to this population [9–12].

To address the need for guidance, the research team considered a patient-centered, multimodal (psychological, physical and pharmacological) approach to the management of osteoarthritis pain. In prior research, the team developed and validated an evidence-informed, paper-based clinical decision support (CDS) tool to guide HCPs in managing osteoarthritis pain in hemodialysis settings (See Supplementary figure 1). This tool was developed based on literature searches and expert opinion and validated by interviewing nephrology and pain management clinicians through multiple rounds of interviews. Details of tool development and validation have been published elsewhere [13].

CDS tools encompass diverse resources, such as algorithms and care pathways, presenting point-of-care knowledge to HCPs. Available in paper and computerized formats, CDS tools are intended to aid HCPs in various aspects of patient care, including optimizing treatment [14, 15]. CDS tools designed to assist clinicians with adjusting medication doses [15], monitoring therapeutic drug levels [15], or implementing evidence-based therapies [16] have been assessed in the context of kidney disease. Overall, the use of CDS tools was associated with enhanced clinician performance in both hospital and outpatient settings, among different populations affected by kidney disease, including patients with end-stage kidney disease [15].

Despite proven utility in improving care, the uptake of CDS tools in practice has been historically poor [17, 18]. Research shows that attractive CDS designs, clinician engagement with the tool, and perceived usefulness to skill development and user confidence facilitate CDS tool use [19]. However, the lack of resources and training, poor CDS applicability in practice, and time constraints have hindered CDS adoption [19].

Improving the uptake of CDS tools involves the application of strategies to address the barriers and facilitators to tool uptake in a given context [20]. Therefore, to facilitate the future goal of implementing the CDS tool across Canadian hemodialysis settings, this study aimed to explore barriers and facilitators that HCPs may face to using the CDS tool for osteoarthritis management, specifically in hemodialysis units.

## Theoretical framework

Implementing new interventions such as CDS tools requires changes in the “individual and collective behaviour” of the relevant actors [21]. Changing professional behaviour is a multilevel process that is facilitated by an “understanding of the influences on behaviour in the context in which they occur” [21]. Therefore, the Theoretical Domains Framework (TDF) was used to develop the interview guide to identify influences on the uptake of the CDS tool by HCPs in the hemodialysis setting [21] (See supplementary file 1 for the interview guide).

## Materials and methods

### Design

This study used a qualitative descriptive approach involving one-on-one, virtual semi-structured interviews. Qualitative description focuses on the participants own words rather than an interpretation of their meanings and was chosen to facilitate a straight description of HCPs’ perceived barriers and facilitators [22]. The research team consisted of two pharmacists with experience working in the hemodialysis setting (the primary researcher and the second coder), a hemodialysis pharmacist (the primary investigator) and a nephrologist with extensive experience caring for seniors with kidney disease. To enhance rigour, the research team followed the Consolidated Criteria for Reporting Qualitative Research (COREQ) [23]. Ethics approval was obtained from the University Health Network (23–5060) and the University of Toronto’s research ethics boards (00044456).

### Sample/setting

HCPs were recruited from April 2023 to June 2023. Inclusion criteria included English-speaking, practicing, nephrologists, nurse practitioners and pharmacists with expertise in hemodialysis. Expertise was defined as experience providing full-time, direct patient care to patients on hemodialysis for a minimum of 1 year. Non-English-speaking HCPs were excluded to ensure feasibility given the limited resources available to this study. Additionally, trainees, residents, and fellows were excluded to ensure participants had sufficient expertise to inform the study aim.

Eligible participants were selected from academic and community settings using a combination of purposeful and snowball sampling techniques. Specifically, the primary investigator forwarded the study invite to potential participants via email and interested persons were instructed to contact the research team for further information about the study. Consent was obtained in writing by the primary researcher prior to conducting the interviews. Consenting participants were asked to relay study information to other clinicians who may be interested in the study. To avoid homogeneity of responses, recruitment from a single institution was limited to two participants and distinguished between nephrologist or nurse practitioner and pharmacist. All those contacted agreed to take part in the study.

### Data collection and analysis

Interviews were conducted by the primary researcher. There was no prior relationship between the primary researcher and the study participants. The interview guide was developed by the primary researcher with insight from the nephrologist. The guide was piloted with the second coder and revised accordingly. Each participant was interviewed once with the presence of only the primary researcher and the participant. Interviews lasted approximately thirty minutes and were audio-recorded and transcribed using Microsoft Teams. The primary researcher conducted the interviews from home.

Data collection was iterative and continued until data saturation (i.e., no new themes of barriers and facilitators emerged with the addition of data). Based on the work of Francis et al, the minimum sample size chosen a priori was 10 [24]. Following the completion of 10 interviews, each additional interview was analyzed for new themes. Sampling ceased after 11 interviews. Throughout the study period the interviewer maintained a reflexive journal to document thoughts or biases impacting the research.

**Fig. 1:**
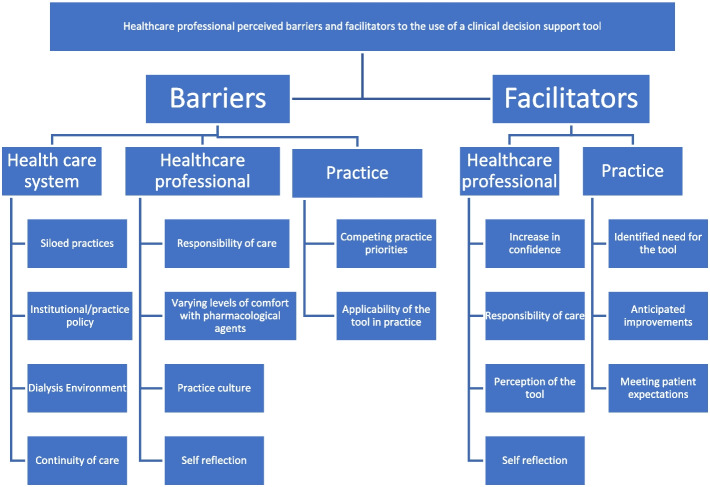
Codes. hierarchical structure of categories and subcategories (codes). This figure represents a hierarchical coding tree of categories and subcategories (codes) that emerged from the data

Data was coded and analyzed inductively to allow the codes and themes to emerge directly from the raw data [25]**.** Coding was conducted by the primary researcher by reading each transcript several times for general impressions. Transcripts were then coded using descriptive codes [26]. A hierarchical coding tree was iteratively developed and refined as themes emerged (see Fig. 1 for a hierarchical structure of categories and subcategories). The second coder independently coded the first 3 transcripts and met with the primary researcher several times until consensus was reached on the codes and themes. The transcripts were coded using Microsoft Word.

## Results

In total, 11 interviews were completed with 3 nephrologists, 2 nurse practitioners and 6 pharmacists from academic and community settings across 5 provinces in Canada. Most participants were between 35–44 years of age and had not attained specialized training in pain management (See Table 1 for participant characteristics).

**Table 1:** Participant characteristics

**Age group**	***n*** **= 11 (%)**
25–34	1 (9)
35–44	6 (54)
45–54	3 (27)
55–65	1 (9)
**Gender**	
Female	6 (54)
Male	5 (45)
**Profession**	
Nephrologist	3 (27)
Nurse practitioner	2 (18)
Pharmacist	6 (54)
**Years of practice**	
1–5	3 (27)
6–10	3 (27)
11–15	2 (18)
> 15	3 (27)
**Practice setting**	
Academic	5 (45)
Community	6 (54)
**Practice location**	
Alberta	2 (18)
British Columbia	1 (9)
Nova Scotia	2 (18)
Ontario	4 (36)
New Brunswick	2 (18)
**Competencies attained in pain management**	1 (9)

**Fig. 2:**
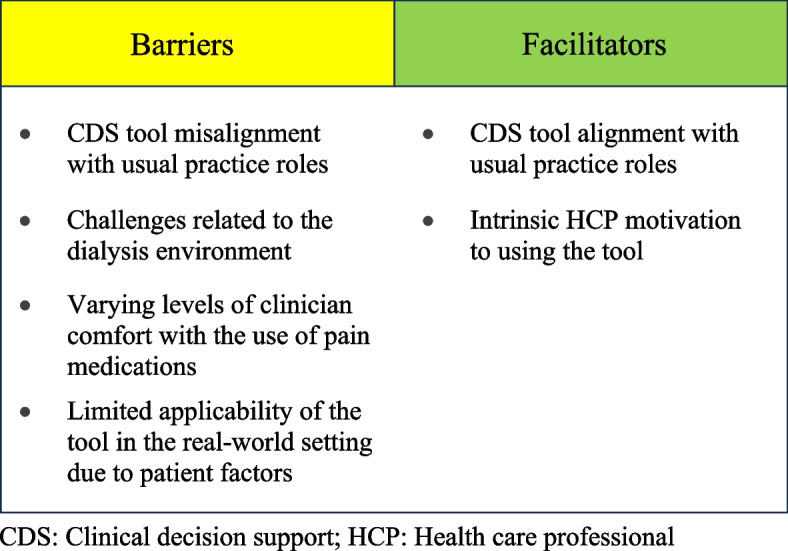
Barriers and facilitators. Main barriers and facilitators to implementing the clinical decision support tool in practice. This figure represents the 4 themes of barriers and 2 facilitators to clinical decision support tool uptake that emerged from the data

Overall, 4 themes of barriers and 2 facilitators emerged (See Fig. 2). Below is a detailed description of each theme.

### Barriers to CDS tool uptake

#### Theme 1: CDS tool misalignment with usual practice roles (main theme)

Many participants viewed themselves as “kidney specialists” and described osteoarthritis pain management as a general medicine issue that did not belong in the realm of kidney care. Participants discussed various factors that discouraged them from adopting the responsibility for managing non-kidney related issues.

Participants spoke of competing practice priorities in the hemodialysis setting and worried about the risk of compromising dialysis care if they become the “dumping ground for all general medicine-related issues”.

P9/Nephrologist: “I know it’s an issue for patients, we could do better, but the question then becomes if we are spending time and effort on osteoarthritis, what are we not spending time and effort on? Is it detracting from care in other ways? That’s always a possibility as these patients have other issues related to general medicine”.

Participants further discussed a lack of training as a hindrance to providing osteoarthritis management.

P9/Nephrologist: “As internal medicine specialists and nephrologists we are not trained in the management of osteoarthritis during our training, the scope tends to be more family practice, so we do tend to rely on our family practice colleagues to deal with this issue”.

Some participants discussed the need to abide by institutional policies or practice cultures that discouraged the management of non-kidney related issues. Participants working in settings where the delivery of primary care was not a practice norm preferred to conform with the established boundaries of care. For example, when participants were asked if managing osteoarthritis was an appropriate part of their jobs, several thought it was, but considered the tool irrelevant to their practices because the nephrologists at that practice site were not addressing issues related to general medicine:

P4/Nurse practitioner: “I know from my interactions with our nephrologists…that they're trying to distance themselves from general medicine and they just want to try to continue to keep it as a specialty area of kidney care… I find the hindrances is keeping up with my colleagues …it's not that I need their blessing and approval but at the same time you kind of want to play ball in the same sandlot with your colleagues…If I'm doing something outside the parameters there will be questions”.

P5/Pharmacist: “It's [the management of osteoarthritis] not super relevant because we have a policy that the rounding MD does not prescribe pain medications”.

Though nephrologists were perceived as central actors to the uptake of the tool, participants expressed that influencing them to deliver primary care without the involvement of a primary care provider (PCP) was unlikely. In fact, two out of three nephrologists who participated in this study strongly felt that the management of non-kidney related issues should default to the PCP.

While a few participants expressed their willingness to coordinate osteoarthritis pain management with PCPs, they explained that siloed practices and poor role definitions for nephrologists and PCPs precluded this arrangement as it was difficult for nephrologists to determine “who is owning that ship” of primary care management.

P4/Nurse practitioner: “I feel the nephrologist and myself are happy to assist, but I don't think we have the sense of reassurance that this is being kind of seen by two sets of eyes type of thing. I feel like once it's on our court everyone kind of just backs up and assumes that we look after it. I think that the hard part is making sure everyone is working together. Unfortunately, the transparency isn't quite there… now it's all individual approaches with us giving a referral or our recommendations back to the other healthcare providers but there is no feedback coming this way as a two-way dialogue.”

These findings indicate a lack of clearly defined roles and responsibilities among hemodialysis teams which may limit implementation of the CDS tool. Findings also highlight the need for improved collaboration between hemodialysis and primary care teams.

#### Theme 2: Challenges related to the dialysis environment (minor theme)

Several aspects of the dialysis environment were viewed as barriers to the successful uptake of the tool, including difficulties in accessing the tool, a lack of human resources to apply the tool and time constraints.

P11/Nephrologist: “I feel like unless the physicians have this printed out somewhere its unlikely they are going to look at it truthfully”.

P1/Pharmacist: “We are, you know a growing program, unfortunately we have a limited number of resources. We are in a community hospital as opposed to a teaching hospital where you have fellows and whatnot, so lots and lots of staff on site to manage these things”.

P8/Nurse practitioner: “Time management would be a big thing for us because really our roles are kind of structured to focus more hemodialysis specific concerns, so I would say kind of incorporating it [the tool] into our time management.”

These barriers illustrate the significance of addressing contextual challenges across dialysis units to promote tool uptake.

#### Theme 3: Varying levels of clinician comfort with the use of pain medications (minor theme)

Several participants reported low levels of comfort using some of the pharmacological agents proposed by the tool. For example, concerns of potential toxicity arose with the use of topical diclofenac and a lack of experience was cited as an obstacle to prescribing cannabinoids and buprenorphine:

P1/Pharmacist: “Some nephrologists just don't like the idea of a topical diclofenac because of again that potential systemic absorption even though it's theoretical… Topical cannabinoids that's another realm that we don't go down because again we're not very knowledgeable on that”.

P3/Pharmacist: “We probably do have practitioners which would avoid the oral NSAIDS at all costs and may be a bit more hesitant with topical diclofenac… we don't tend to use much Suboxone either or the Butrans patch.”

Opioid prescribing in general came under scrutiny with variations in prescriber comfort level:

P10/Pharmacist: “There is one nephrologist that will prescribe opiates for pain but the other three will not…a patient might get a prescription for hydromorphone but then the patient runs out and the other nephrologist isn't willing to prescribe”.

Notably, a few participants indicated a readiness to learn about non-conventional pharmacological approaches for pain management:

P7/Nephrologist: “If there are options that are effective, particularly in the dialysis population, because there's limits to what we can use in terms of agents as well as dosing regimens. So I guess just understanding and the awareness of the available options would be always appreciated”.

P2/Pharmacist: “ My comfort level with suboxone is such that I wouldn’t necessarily be recommending it until I become more comfortable with it”.

This finding points to variations in prescribing patterns both within and across dialysis centers. Most participants preferred using a familiar drug and highlighted the need for educational initiatives to increase clinician comfort level with some of the proposed analgesics.

#### Theme 4: Limited applicability of the tool in the real-world setting due to patient factors (minor theme)

A few participants questioned the general applicability of the tool to the hemodialysis population and anticipated that some patients would not fall within the outlined treatment cascade, such as those who had reached the narcotic level. Participants also noted that many dialysis recipients had low socioeconomic status and the cost of the recommended treatments (topical agents and ancillary services) would be beyond their reach:

P9/Nephrologist: “The hospital-based physio and OT there is a big waiting list and if patients don't have coverage for private, it certainly limits access and delays access, and so then we know we tend to jump to pharmacologic treatments”.

### Facilitators to CDS tool uptake

#### Theme 1: CDS tool alignment with usual practice roles (minor theme)

Although many participants reported misalignment of the tool with their practices, a few participants felt the tool was well aligned with their roles. These participants perceived themselves as “providers of holistic care” and viewed the management of osteoarthritis pain as an aspect of comprehensive care that should be provided by dialysis teams.

For example, when participants were asked if osteoarthritis pain management was an appropriate part of their jobs one participant stated:

P7/Nephrologist: “Realistically yes, because we're already seeing them on a weekly basis, it [osteoarthritis] becomes something we end up having to manage”.

Pharmacists considered themselves well positioned to provide recommendations for osteoarthritis management across hemodialysis and primary care teams due to their strong capacity for monitoring and sharing information:

P2/Pharmacist: “Prescribers being their family doctors are resistant to prescribe something in a complex hemodialysis patient, and therefore ask us for recommendations…we are in a good position where we can monitor efficacy for any type of therapeutic intervention. Pain is one of those things because we start that intervention, we see that it is started through the family doctor or whoever is ordering it, but then we can follow up and monitor for adverse effects”.

These participants also recognized the increasing trend of patients without PCPs and indicated their willingness to adopt the sole responsibility of osteoarthritis pain management in the absence of other care:

P8/Nurse practitioner: “We're having less patients with primary care providers, so therefore we are finding that prescribers within the hemodialysis world are taking on more and more responsibilities for primary care and management needs. I think we're looking at providing holistic care. I do think it's a big portion of care that we are able to provide”.

The finding of tool alignment with existing roles reveals a readiness for tool uptake among select hemodialysis teams.

#### Theme 2: Intrinsic HCP motivation to using the tool (main theme)

Acknowledging the rigours of dialysis treatments and their negative impact on patients’ quality of life, as well as the preferences of patients to receive primary care by hemodialysis teams due to convenience, several participants expressed a desire to prioritize the management of osteoarthritis. Participants explained that providing primary care during dialysis treatments may help patients cope with the burden of their disease state by reducing the number of visits required to manage their comorbidities.

P4/Nurse practitioner: “I wish we all kind of revamped kind of the way we look at patients and I think whether it's osteoarthritis or renal or whatever we do, look at it in a timely fashion just to break down barriers 'cause they are coming in three times a week. So for us to say go see your family doctor and now the other days that they're off have to be filled with another medical appointment. I don't think is a service for them, it looks at quality of life.”

Participants perceived the use of the tool could affirm better practices through standardizing care and prompting clinicians to re-evaluate therapy which would lead to improvements in patient care.

P7/Nephrologist: “If you have a tool that puts all of us on the same page and we all agree upon and this looks really good truthfully, I think it would standardize nephrologists, nurse practitioners and pharmacists in a guideline way”.

P2/Pharmacist: “Oftentimes, I find that there’s this therapeutic inertia, that we just keep it going and I find that this tool actually suggests and actually encourages the reassessment and actually tapering and removing therapy if it’s not being efficacious”.

Additionally, participants believed the tool would increase their confidence and empower their decision making to manage osteoarthritis pain.

P2/Pharmacist: “You are not kind of floundering around, it gives you one basis point, it's good to have kind of a lighthouse that you are aiming for and that's what it does, provides like a good recommendation. It's offering things that I definitely haven't considered which is great”.

Participants also highlighted that increased collaboration between hemodialysis clinicians and PCPs can promote better osteoarthritis pain management for patients.

P5/Pharmacist: “If there is collaboration with the GP [general practitioner], if the GP starts an intraarticular steroid or an opioid, you know instead of the patient having to go to the GP …possibly its followed on the dialysis unit by a pharmacist or a nurse”.

The emergence of this theme exposes HCPs’ motivations for using the tool to improve patient care in the hemodialysis setting and confirms the need for collaboration among hemodialysis and primary care teams.

## Discussion

TDF-informed qualitative interviews with HCPs revealed several barriers and facilitators to the use of a CDS tool within hemodialysis units in Canada. To the research team’s knowledge, this is the first study to explore the perceptions of HCPs on the use of a decision aid for osteoarthritis pain management in this setting. Barriers included challenges related to the dialysis environment, varying levels of clinician comfort with pain medications, and limited applicability of the tool in the real-world. Tool alignment with practice roles emerged as both a barrier and facilitator to tool uptake. HCP motivation for using the tool to improve quality of care among hemodialysis patients emerged as a key facilitator. Addressing these barriers and facilitators may promote effective implementation of the tool and improve osteoarthritis pain management among hemodialysis patients.

This research supports the findings of Presseau et al, which identified that the alignment of a new intervention—such as individualized dialysate temperature—with established practice roles was both a barrier and a facilitator to adoption among hemodialysis clinicians [27]. The authors also noted that intervention uptake was influenced by “sequences of clinical behaviours”, complex professional interactions where the “behaviour of one profession, defined by their professional role, serves as a social influence to the other profession” [27]. In the present study, participants’ perception of CDS tool alignment with existing practice roles strongly influenced their attitudes toward tool adoption. Participants identifying as “kidney specialists” reported the misalignment of the tool with their practice roles and resisted its adoption. In contrast, those identifying as “providers of holistic care” found the tool well-suited to their practices and were amenable to its adoption. “Sequences of clinical behaviors” were evident in this study as the actions of nephrologists clearly influenced the behaviours of both nurse practitioners and pharmacists who consistently reported a need to conform to the nephrologist’s approach to care. To increase tool uptake, initial implementation efforts should target select institutions where the provision of primary care is aligned with existing roles since clinicians working in these institutions would be more accepting of a tool for osteoarthritis management.

The study findings align with considerable literature emphasizing that poor communication between nephrologists and primary care physicians limits nephrologists' ability to deliver primary care services [28–31]. In the present study, participants expressed that “siloed practices” and the inability to ascertain who is “owning that ship” of primary care management dissuaded nephrologists from engagement in primary care. Other barriers found in this study including environmental challenges, HCP discomfort with pain medications, and limited tool applicability also align with prior research. Beers et al cited lack of time, limited knowledge and lack of infrastructure as challenges to nephrologist directed primary care [30]. Further, in a qualitative systematic review and meta-aggregation of barriers and enablers to implementing and using CDS systems for chronic diseases, Chen et al identified lack of time, limited resources and lack of applicability due to patient factors as barriers to CDS system uptake [19].

This research offers novel findings on the motivations of hemodialysis clinicians for incorporating a CDS tool into their practices as part of comprehensive care. Although participants acknowledged several factors which precluded tool alignment with existing practice roles including competing priorities, lack of training, institutional policies, and siloed practices among different health teams, a few indicated a willingness to adopt the tool into their practice to improve the quality of osteoarthritis management and the patient’s quality of life. While motivation may help promote tool uptake, it cannot resolve implementation barriers identified. Strategies to overcome these barriers are crucial to tool uptake.

Notably, several clinicians who participated in this research felt that collaborations between hemodialysis teams and PCPs were a requirement for tool adoption because of the complexity of renal failure and the various practice demands of the dialysis environment. Thus, enhancing collaboration between hemodialysis and primary care teams through ensuring clearly defined roles and responsibilities among them would help nephrologists determine the scope of their primary care responsibilities and may encourage their participation in the provision of care.

The effectiveness of implementation may also be enhanced by mobilizing educational initiatives such as continuing educational programs to increase clinician knowledge and comfort with pain medications. Previous research has demonstrated improvements in “clinician knowledge, attitudes, and pain practice behavior” following a continuing education program although a sustained effort at educating clinicians over an extended period of time may be required to change attitudes regarding pain management [32, 33]. Further, given the time constraints and lack of resources that exist within dialysis settings, the allocation of additional human resources, such as nurses, to facilitate the application of the tool should be considered. In a recent study examining PCP experiences with using a CDS tool for pain management, participants recommended expanding tool access to clinical staff to better integrate the tool into the workflow and address clinician time constraints [34].

This study is strengthened by a diverse participant pool that varied by clinician type and practice location. Considering the potential for practice variations across different sites, it is crucial to gather insights from a wide range of perspectives rather than focusing on HCPs in a single geographical area. Also, the value of each participant was maximized by limiting recruitment from a single institution to two participants. The study has several limitations that should be noted. First, we focused on HCPs’ perspectives since they are the main users of CDS tools although decision aids impact both HCPs and patients, and having patient support behind the tool may influence implementation outcomes. Second, we did not purposively select participants from practices that delivered primary care. Therefore, some participants were not as information rich because osteoarthritis management was not relevant to their practices.

## Conclusion

Hemodialysis teams are amenable to the use of a CDS tool for managing osteoarthritis pain, however, successful implementation depends on tool alignment with practice norms. Widescale implementation may become feasible with increased collaboration among hemodialysis and primary care teams. Future research should explore the perceptions of PCPs using the tool in collaboration with hemodialysis teams.

### Supplementary Information

Supplementary Material 1: Supplementary file 1. Interview guide

Supplementary Material 2: Supplementary figure 1. Clinical decision support tool. Algorithm for patients on hemodialysis with pain associated with osteoarthritis. This figure represents the final version of the tool for the management of osteoarthritis pain in the hemodialysis population following validation and revisions

## Data Availability

The datasets used and/or analysed during the current study (interview transcripts) are not made publicly available to protect the identity/privacy of study participants. Portions of the data generated or analysed during this study are included in this published article.

## Abbreviations

HCPs: Healthcare professionals
CDS: Clinical decision support
TDF: Theoretical Domains Framework
PCPs: Primary care providers
