# Supplementary material for: Barriers and facilitators to using a clinical decision support tool for the management of osteoarthritis pain in patients undergoing hemodialysis: a qualitative study
Source: BMC Prim Care. 2024 Aug 19;25:308. doi: 10.1186/s12875-024-02564-5 (PMC11331684; doi:10.1186/s12875-024-02564-5)
Supplement: Supplementary file 1 — Supplementary Material 1: Supplementary file 1. Interview guide [file 12875_2024_2564_MOESM1_ESM.docx]

Additional supplementary file 1: Interview guide

**Professional Role**

1. **How relevant is the management of osteoarthritis pain to the scope of your practice?**
2. Do you think it’s an appropriate part of your job to manage osteoarthritis pain?
3. Is there anything else about your professional role in the dialysis suite that influences your ability to manage osteoarthritis pain in this population?

**Beliefs about capabilities**

1. **How confident are you in the management of osteoarthritis pain without a clinical decision support tool**
2. If low confidence, what needs to happen to increase your confidence?
3. How might the use of the clinical decision support tool affect your confidence?

**Knowledge**

1. **In your practice do you rely on any type of guidance when managing osteoarthritis pain?**
2. If yes, what type of guidance do you use? Why do you use it? What do you think of it?
3. If no, can you explain why?
4. Do you follow up to monitor patients progress after initiating therapy for osteoarthritis?
5. If yes, how do you monitor? What is the outcome usually? If no, can you explain why?

**Skills**

1. **How easy or difficult is it to manage osteoarthritis pain without a clinical decision support tool?**

a- How much expertise or experience do you think one needs to have to effectively manage osteoarthritis pain?

b-How would the use of the tool influence your ability to manage osteoarthritis pain in your practice?

**Environmental context and resources**

1. **What aspects of your practice environment help or hinder the use of this tool?**
2. How much would the systems in place support or hinder the use of the tool?
3. What needs to happen in your environment to help you use the tool?

**Social influences**

1. **How might views/opinions of others (colleagues, patients, professional groups) influence your decision to use the tool?**

**Emotions**

1. **We know that clinicians’ comfort level can affect their practice. For example, you might feel uncomfortable about prescribing an opiate for a patient who is already prescribed a number of drugs, or you might get some job satisfaction from knowing that you’ve taken action to alleviate the patient’ pain condition. When are you managing osteoarthritis pain in your practice what feelings arise for you?**
2. How would using the tool impact those feelings?

**Behavioral regulation**

1. **Are there situations that help prompt you to use the tool?**
2. Are there particular types of patients for whom the use of the tool is considered especially relevant/irrelevant or more easy or difficult?
3. Can you elaborate?

**Beliefs about consequences**

1. **What do you think are the consequences of or disadvantages in managing osteoarthritis pain without a clinical decision support tool?**
2. How would the use of the CDS tool influence these consequences?

**Motivation**

1. **How much do you want to use the tool in your own practice?**
2. How much benefit do you perceive in using this tool?
3. What kind of additional information would most likely influence your decision to use the tool?
4. What needs to happen for you to utilize the tool in your practice?

Are there other factors about this topic that you think might be important that we have not covered?
