# Supplementary material for: Barriers and facilitators to using a clinical decision support tool for the management of osteoarthritis pain in patients undergoing hemodialysis: a qualitative study
Source: BMC Prim Care. 2024 Aug 19;25:308. doi: 10.1186/s12875-024-02564-5 (PMC11331684; doi:10.1186/s12875-024-02564-5)
Supplement: Supplementary file 2 — Supplementary Material 2: Supplementary figure 1. Clinical decision support tool. Algorithm for patients on hemodialysis with pain associated with osteoarthritis. This figure represents the final version of the tool for the management of osteoarthritis pain in the hemodialysis population following validation and revisions [file 12875_2024_2564_MOESM2_ESM.pdf]

Algorithm for patients on haemodialysis (HD) with pain associated with osteoarthritis (OA)

Assessment and screening

This pathway is not intended for use in the following cases:
Inflammatory arthritis, Septic arthritis, Monoarticular arthritis, Infection, Inflammation, Hip/knee replacement, Previous fracture, Crystal arthropathies, Tumour (history of cancer, unexplained weight loss, significant night pain, severe fatigue).[1,2]

Likely pain related to OA if: [1]

- Pain in any joint, but usually in knees, hips, or hands
- Characterised by stiffness, discomfort, joint function impairment
- Joint pain lasting < 30 minutes upon waking
- Joint pain generally related to end of day pain/activity/overuse

- **Assess baseline pain and functional interference.** Click here for the Brief Pain Inventory [3]
- **Screen for psychosocial symptoms of pain**.[4] Click here for screening tool (ECHO)[5]
  - If patient screens + for psychosocial symptoms ***REFER*** to social worker, psychologist, or nephrology dedicated psychiatry as needed.
  - Click here for free mental health resource (Bounce Back Ontario)[6]
    Skill building program delivered via phone with a coach and/or via online videos.

Basic principles of management

Combination of treatment modalities including nonpharmacological and pharmacological therapies is strongly recommended. Set SMART [7] goals and individualize therapy. Provide a culturally safe environment.[8]

Step 1: Non-pharmacological therapy

- **Protective modalities**: Assess position of joints during HD (keep joints in a neutral position), reduce stress on joints during sleep (firm mattress, pillow between legs)[1], use raised beds and toilet seats[1], rest for 30-60 sec every 5-10 min when stretching or moving joints [1]
- **Physical management**: Heat therapy, assistive devices, neuromuscular training, physical exercise, weight management [1]
  REFER to PT/OT as needed. ***REFER*** to dietician/weight loss clinic if obese (BMI≥30kg/m^2)
- **Patient education/ information**: Self-management[9,10], meditation[11,12], sleep hygiene[13]

Step 2: Add a Topical agent if patient is symptomatic and pain is localized

Not covered under ODB, cost may be a barrier

*Preferred **Diclofenac diethylamine (Voltaren emulgel®, Voltaren emulgel ES®) $**	**Methyl salicylate with menthol/ camphor $$**	**Capsaicin cream (Zostrix®, Zostrix HP®) $$**	**Topical CBD/THC $$$**
• 1.16%, 2.32% (2-4 g topically TID-QID)[1,14]<br>• May cause bruising<br>**Diclofenac sodium (Pennsaid®) $$$**<br>• Knee OA 40 drops/knee QID [1] or 50 drops/knee TID	• Hand OA (Topically TID-QID)[1]	• Hand OA/small joints 0.025% or 0.075% BID-QID[1,14]<br>• May take> 2 weeks for onset of action, adherence can be a problem	• Lack of evidence to support its use.[15]<br>• May ***REFER*** to a cannabis clinic for a trial

→ **Combine step 2 and 3 for severe pain**

Monitor therapy in 2-4 weeks
scroll down to monitoring

Step 3: Add an oral agent if patient still symptomatic

NSAIDS

- Short term use may be considered for patients with anuria and without cardiac or gastrointestinal contraindications [2]
- Consider gastric ulcer risk [2]

Acetaminophen

- Published evidence has not demonstrated clinically relevant effects on pain/function [16-18]
- Associated with liver dysfunction and hypertension[19]
- May cause INR fluctuations at doses ≥ 2g/day for several days [20]

Monitor therapy in 2 weeks
scroll down to monitoring

Step 4: Trial an intra-articular corticosteroid if patient still symptomatic

- Intra-articular corticosteroid injections may provide short-term pain relief for Hip/Knee OA [1]
- May be administered by MRP or ***REFER*** to rheumatologist, physiatrist, pain specialist or chronic pain clinic (e.g., TAPMI)[7]

Monitor therapy in 2 weeks, then in 3 months
scroll down to monitoring

Step 5: Add a short-term opioid course to enable non-pharmacological modalities

Opioid management tips

- Do not initiate opioids without an exit strategy. Withdrawal can occur following days, weeks, or months of therapy. May consider tapering by reducing the dose by 5 to 10% of original dose every 2 to 4 weeks.[21,22]
- Ensure there is a single opioid prescriber on the patient's care team who assumes responsibility for adjusting the opioid regimen.
- Titrate slowly based on pain assessment but emphasize improvement in function as a primary goal.
- Opioids have a small effect on chronic pain/physical function with increased side effects (constipation, respiratory depression, fractures, hyperalgesia)[23]. Continue therapy only if there is clinically meaningful improvement in pain and function[24] e.g., 30% improvement from baseline on a 3-item PEG scale [25,26]
- Consider a prophylactic bowel regimen to manage constipation (senna glycosides +/-PEG+/- lactulose).[2]
- Co-prescribe naloxone for at-risk patients (high opioid dosages ≥ 50MME/day, concurrent benzodiazepine use, history of overdose, history of substance use disorder, during tapers or for patients residing with children who are at risk of accidental poisoning.[27]
- **Opioid resources: Naloxone education [28], Opioid conversion table[29], Opioid manager[30], Opioid guideline for chronic non cancer pain [31]**

Avoid Morphine and Meperidine [14]

Buprenorphine

- Favourable side effect profile (less risk of respiratory depression, tolerance, hyperalgesia, minimal renal elimination)[32]
- Special considerations for managing acute/postoperative pain in patients receiving buprenorphine therapy [33]
- If patient is switching from an opioid, ***REFER*** to pain specialist for buprenorphine induction[34]
- If patient is **not** on opioids, may initiate buprenorphine as follows:

SL formulation buprenorphine/naloxone (Suboxone®) [35-37]
2mg initially, then up titrated depending on patient's needs.
Maximum suggested daily dose of 16-24mg.
Pain control may be optimized with BID dosing.

♦ General benefit under ODB (available in 2/0.5mg, 8/2mg)

Transdermal patch (Butrans®) [38]
5-10mcg/h patch Q7 days. Dose can be **increased Q7 days**. Max dose 20 mcg/h Q7 days.[14]

♦ Not a benefit under ODB (available in 5,10,15,20 mcg/h patches)**$$$**

Hydromorphone

- Neurotoxic H3G accumulates if dialysis is discontinued [14,39,40]
- **IR** preferred to minimize the risk of accumulation.

Initial IR dosing: 0.5mg to 1mg PO Q3-4 hours PRN [2,14]
Extending the dosing interval is recommended [40] (e.g., Q6-8 h)

♦ General benefit under ODB

CR dosing: PO Q12 hours [14]

♦ General benefit under ODB (3mg, 4.5mg, 6mg, 9mg, 12mg, 18mg)
♦ Require EAP (24mg & 30mg)

Methadone

- It is of clinical value in the HD population.
- It demonstrates a complex pharmacokinetic profile [41]
- ***REFER*** to an experienced methadone prescriber (pain specialist, chronic pain clinic or palliative care)

♦ Only 10mg/ml oral liquid concentrate is covered by ODB

Monitor therapy in 2-4 weeks, monitor weekly with dose escalations and tapers
Scroll down to monitoring

Monitoring

- Pain reduction, Functional outcomes, Pain coping. Click here for the Brief Pain Inventory [5]
- Aberrant opioid use
- Medication related side effects

Worsening symptoms

- Assess patient compliance and /or comprehension gaps
- ***REFER*** to pain specialist, chronic pain clinic, rheumatologist or orthopedic surgery as required. (Consider imaging when referring to rheumatology/orthopedic surgery)[1]
- May ***REFER*** to palliative pain clinic depending on institutional policy.

No change in symptoms

- Review exercise/ activity and avoid overuse. Schedule frequent breaks and recovery positions.
- Review individualized goals, medication regimen, and consider alternative therapeutic drug/drug class
- May ***REFER*** to outpatient rehabilitation, pain specialist or chronic pain clinic
- Consider imaging if failure to respond to evidence-based management over a 12-week period[1]

Improvement in symptoms

- Reassess medication regimen as needed
- Engage in physical and self management modalities
- Reinforce exercise (as appropriate)
- Gradually increase exercise to achieve individualized goals

1. Osteoarthritis tool: Canada: The Arthritis Alliance of Canada, the Centre for Effective Practice, the College of Family Physicians of Canada. https://cep.health/media/uploaded/CEP_OATool_2017.pdf. Published 2017. Accessed January 31 2024
2. Ontario renal network (ORN). Pain resource. https://www.ontariorenalnetwork.ca/sites/renalnetwork/files/assets/PainResource_0.pdf. Published 2019. Accessed January 31 2024
3. Project ECHO at University Health Network. Resources. Chronic Pain. Brief pain inventory, https://uhn.echoontario.ca/Resources/Chronic-Pain. Accessed January 31 2024
4. Adams LM, Turk DC. Psychosocial factors and central sensitivity syndromes. *Curr Rheumatol Rev*. 2015; 11: 96–108
5. Project ECHO at University Health Network, ECHO psychosocial screening interview guide, https://uhn.echoontario.ca/Resources/Psychosocial-Resources. Accessed January 31 2024
6. BounceBack Ontario – Canadian Mental Health Association, Ontario, https://bouncebackontario.ca/. Accessed January 31 2024
7. Toronto Academic Pain Medicine Institute (TAPMI), https://tapmipain.ca/healthcare-practitioner/. Accessed January 31 2024
8. Murphy L, Ng K, Isaac P, et al. The Role of the Pharmacist in the Care of Patients with Chronic Pain. *Integr Pharm Res Pract*. 2021; 10: 33–41
9. Arthritis Society of Canada. Osteoarthritis Self-Management - Exercise, Diet, Pain management, https://arthritis.ca/about-arthritis/arthritis-types-(a-z)/types/osteoarthritis/osteoarthritis-self-management. Accessed January 31 2024
10. Dr. Andrea Furlan - YouTube, https://www.youtube.com/c/DrAndreaFurlan. Accessed January 31 2024
11. Calm, https://www.calm.com/. Accessed January 31 2024
12. Lagunju O, Bahl S, Staff M, et al. Mindful, https://www.mindful.org/. Accessed January 31 2024
13. Wilbanks J. Healthy Sleep, https://sleepeducation.org/healthy-sleep/. Accessed January 31 2024.
14. BC Renal agency. BCPRA Guidelines and Drug Choices for Chronic Pain in Dialysis Patients, http://www.bcrenal.ca/resource-gallery/Documents/Guidelines%20and%20Drug%20Choices%20for%20Chronic%20Pain%20in%20Dialysis%20Patients.pdf. Published 2017. Accessed January 31 2024
15. Health Quality Ontario. Quality Standards. Osteoarthritis. https://www.hqontario.ca/Portals/0/documents/evidence/quality-standards/qs-osteoarthritis-clinician-guide-en.pdf. Published 2018. Accessed January 31 2024
16. National Institute for Health and Care Excellence (NICE). Guidance. Osteoarthritis: care and management. https://www.nice.org.uk/guidance/cg177. Published 2022. Accessed January 31 2024
17. Leopoldino AO, Machado GC, Ferreira PH, et al. Paracetamol versus placebo for knee and hip osteoarthritis. *Cochrane Database Syst Rev*. 2019; 2: CD013273
18. da Costa BR, Reichenbach S, Keller N, et al. Effectiveness of non-steroidal anti-inflammatory drugs for the treatment of pain in knee and hip osteoarthritis: a network meta-analysis. *Lancet*. 2017; 390: e21–e33.

19. MacIntyre IM, Turtle EJ, Farrah TE, et al. Regular Acetaminophen Use and Blood Pressure in People With Hypertension: The PATH-BP Trial. *Circulation*. 2022; 145: 416–423.
20. Hughes GJ, Patel PN, Saxena N. Effect of acetaminophen on international normalized ratio in patients receiving warfarin therapy. *Pharmacotherapy*. 2011; 31: 591–597.
21. Murphy L, Babaei-Rad R, Buna D, et al. Guidance on opioid tapering in the context of chronic pain: Evidence, practical advice and frequently asked questions. *Can Pharm J (Ott)*. 2018; 151: 114–120
22. CDC. Guidelines for prescribing opioids for chronic pain. Pocket Guide: Tapering opioids for chronic pain. https://www.cdc.gov/drugoverdose/pdf/clinical_pocket_guide_tapering-a.pdf. Accessed January 31 2024
23. Ivers N, Dhalla IA, Allan GM. Opioids for osteoarthritis pain: benefits and risks. *Can Fam Physician*. 2012; 58: e708
24. Dowell D et al. CDC Clinical Practice Guideline for Prescribing Opioids for Pain — United States, 2022. MMWR Recomm Rep 2022;71(No. RR-3):1–95.
25. Ostelo RWJG, Deyo RA, Stratford P, et al. Interpreting change scores for pain and functional status in low back pain: towards international consensus regarding minimal important change. *Spine (Phila Pa 1976)*. 2008; 33: 90–94
26. Krebs EE, Lorenz KA, Bair MJ, et al. Development and Initial Validation of the PEG, a Three-item Scale Assessing Pain Intensity and Interference. *J Gen Intern Med*. 2009; 24: 733–738.
27. CDC. Guidelines for prescribing opioids for chronic pain, https://www.cdc.gov/drugoverdose/pdf/prescribing/Guidelines_factsheet-a.pdf. Accessed January 31 2024
28. Ontario College of Pharmacists (OCP). Resources on Naloxone and How to Obtain Naloxone Kits, https://www.ocpinfo.com/naloxone-resources/. Published 2017. Accessed January 31 2024
29. Mosca L. Machealth. Opioid conversion table - Safer opioid prescribing strategies, https://machealth.ca/search?q=conversion%20table. Accessed January 31 2024
30. Centre for Effective Practice.Toronto. Opioid Manager. https://cep.health/media/uploaded/CEP_Opioid_Manager_2017.pdf. Published 2017. Accessed January 31 2024
31. Busse JW, Craigie S, Juurlink DN, et al. Guideline for opioid therapy and chronic noncancer pain. *CMAJ*. 2017; 189: E659–E666.
32. Johnson RE, Fudala PJ, Payne R. Buprenorphine: considerations for pain management. *J Pain Symptom Manage*. 2005; 29: 297–326
33. Warner NS, Warner MA, Cunningham JL, et al. A Practical Approach for the Management of the Mixed Opioid Agonist-Antagonist Buprenorphine During Acute Pain and Surgery. *Mayo Clin Proc*. 2020; 95: 1253–1267
34. Powell VD, Rosenberg JM, Yaganti A, et al. Evaluation of Buprenorphine Rotation in Patients Receiving Long-term Opioids for Chronic Pain. *JAMA*. 2021; 4: e2124152
35. Suboxone monograph, https://pdf.hres.ca/dpd_pm/00063411.PDF. Published 2020. Accessed January 31 2024
36. Tobin DG, Lockwood MB, Kimmel PL, et al. Opioids for chronic pain management in patients with dialysis-dependent kidney failure. *Nat Rev Nephrol* 2022; 18: 113–128.

37. Steenhof N, Ng K. Buprenorphine-naloxone in chronic pain: Overcoming stigma for safer opioid management. *Can Pharm J (Ott)*. 2023; 157: 7–9.
38. Butrans monograph, https://pdf.hres.ca/dpd_pm/00035932.PDF. Accessed January 31 2024
39. Paramanandam G, Prommer E, Schwenke DC. Adverse effects in hospice patients with chronic kidney disease receiving hydromorphone. *J Palliat Med*. 2011; 14: 1029–1033.
40. Vondracek SF, Teitelbaum I, Kiser TH. Principles of Kidney Pharmacotherapy for the Nephrologist: Core Curriculum 2021. *Am J Kidney Dis.* 2021; 78: 442–458.
41. College of Physicians and Surgeons of British Columbia. Guidelines Methadone for analgesia, https://www.cpsbc.ca/files/pdf/DP-Methadone-for-Analgesia-Guidelines.pdf. Published 2022. Accessed January 31 2024
